# Evaluation of C-shaped canals in maxillary molars in a Chinese population using CBCT

**DOI:** 10.1186/s12880-022-00831-4

**Published:** 2022-05-29

**Authors:** Yuyan Qian, Yamei Li, Jukun Song, Ping Zhang, Zhu Chen

**Affiliations:** 1 Department of Endodontics, Guiyang Hospital of Stomatology, Guiyang, 550002 Guizhou China; 2 Zunyi Medical University, Zunyi, 563000 Guizhou China; 3 Department of Oral and Maxillofacial Surgery, Guizhou Provincial People’s Hospital, Guiyang, 550002 Guizhou China; 4 Department of Pediatric Dentistry, University of Alabama at Birmingham, Birmingham, AL USA

**Keywords:** Cone-beam computed tomography, C-shaped root canal, Maxillary molars, Southwestern Chinese subpopulation

## Abstract

**Introduction:**

The aim of this study was to evaluate the prevalence and characteristics of C-shaped root canals in maxillary first (MFMs) and second (MSMs) molars in a Southwestern Chinese population using cone-beam computed tomographic (CBCT).

**Methods:**

CBCT images of MFMs (*n* = 1488) and MSMs (*n* = 1547) from 1508 subjects enrolled in Guiyang Hospital of Stomatology between July 2018 to February 2021 were evaluated for the incidence and types of C-shaped root canals. Differences by age, sex, left or right side, and bilateral symmetry were also evaluated.

**Results:**

C-shaped root canals were present in 2.93% MFMs and MSMs (*n* = 3035) in the Southwestern Chinese population. A significant higher incidence was observed in the MSMs (5.24%) than in the MFMs (0.54%). Increased incidences were noted in teeth with fused root. Two major types and 5 subtypes of C-shaped canal system of maxillary molars were defined, and the most common type of C-shaped canals is fusion of mesial-buccal (MB) and distal-buccal (DB) canals (Type I subtype C). No significant gender and age differences were noted in the prevalence of C-shaped root canals in this population, and no significant difference was seen in its incidence in the left or right MFMs and MSMs. The bilateral occurrence was observed in 1.27% of the patients.

**Conclusion:**

C-shaped root canals are more frequently present in MSMs than in MFMs in the Southwestern Chinese population, with Type I subtype C (MB-DB canal fusion) as the most common subtype and low incidence of bilateral symmetry.

## Introduction

The morphology of root canal system is complex and present different anatomical characteristics such as isthmuses between canals, additional root canals, and irregularly shaped canals [1–3]. Comprehensive knowledge of root canal morphology and its possible variations is fundamental for successful root canal treatment (RCT) [4]. Different approaches have been developed to visualize root canal morphology such as magnification devices, conventional periapical films, and cone beam computer tomography (CBCT). The CBCT is a practical tool for analysis the anatomic complexities of the root canal system by providing three dimensional reconstruction images [3].

The RCT of maxillary molars has the highest clinical failure rates, possibly due to high incidence of additional root canal and the presence of fused root canals including C-shaped root canal, and that isthmus in C-shaped canal could not be cleaned completely [5, 6]. In general, a C-shaped root canal is defined as a root canal that in transverse section is shaped like the letter C [6]. However, such root canals are not always continuously C-shaped from orifice to apical foramen. A tooth is therefore usually defined as having a C-shaped root canal system when any arbitrary cross-section presents a C-shaped root canal configuration, which is difficult to clean or filled by RCT. C-shaped root canal is most frequently found in mandibular second molars [5]. However, C-shaped canals may also occur in any molar area, such as mandibular first molars [7], first premolars [8] and maxillary molars [9].

A Southern Chinese subpopulation study [10] recorded 22 variant categories of root canal system of maxillary molars, but C-shaped canals of maxillary molars are not mentioned and classified. Martin et al. [11] classified C-shaped canals of maxillary molars according to root fusions, and three consecutive axial cross sections with a continuous large C-shaped canal system, or a continuous C-shapes with 2 main canal lumen in the extremities connected by a large isthmus, which is based on Fan et al.’s [5] study regarding the lower molars. However, due to the difference in the number of roots and canals and the types of root fusion between the upper and lower molars, the C-shaped configurations could be different. As a result, Fan’s classification of mandibular C-shaped molars [5] cannot be applied to the maxillary molars. A systematic review with meta-analysis [12] showed that no statistical analysis was performed for proportion of C-shaped configuration in maxillary molars because of the limited number of studies. According to the number of root canals and canal merging position, C-shaped canal of maxillary molars was divided into three main types, and subdivided by the root canal which constitutes the ‘C’ shape in a Korean study [9]. This classification is simple, clear and easy to be used in clinic. However, their study only concerned about the incidence and types of C-shapes, differences by age, sex, location in the jaw, and bilateral symmetry were not mentioned. It has been reported that anatomical symmetry or contra-lateral similarity regarding the number of root canals in the maxillary and mandibular molars was as high as 70–81% [13]. If this is the case with the presence of C-shaped canals in maxillary molars, clinicians should pay more attention to the possibility of the presence of C-shaped canals in the contralateral molars if one side of maxillary molars is identified with C-shaped canals. Therefore, in addition to the prevalence and characteristics of the C-shaped canals in maxillary molars, understanding the association of root canal system in contra-lateral maxillary molars is important for endodontic practice.

People from different geographic regions may have different root canal morphology [14]. In this study, we evaluated the prevalence and characteristic of C-shaped canals in maxillary first molars (MFMs) and maxillary second molars (MSMs) in a Southwestern Chinese subpopulation. Differences by age, sex, left or right side, and bilateral symmetry were also analyzed. Understanding the morphology and possible variation of root canals is important for guiding root canal treatment.

## Methods

A total of 3035 CBCT images including 1488 MFMs and 1547 MSMs were obtained from 1508 patients (1201 men and 1834 women) born in Southwestern China who required radiographic examination by CBCT as part of their dental treatment at Guiyang Hospital of Stomatology, Guizhou, China between July 2018 to February 2021. The mean age of the patients was 38.56 ± 14.38 years old. The inclusion criteria were to have at least one MFM or MSM. Teeth with immature apices, apical periodontitis, root resorption, root canal fillings, post or other crown reconstruction were excluded. Cases where the anatomy was compromised by physiological or pathological processes or with unclear root canal morphology were also excluded from the study [15]. The study was approved by the Ethics Committee of Guiyang Hospital of Somatology, and informed consent was obtained from the patients.

All images were taken using a 3D Accuitomo scanner (Morita, Kyoto, Japan) with image capture parameters set at 90 kV and 5.0 mA, and an exposure time of 30 s. To contrast the bilateral teeth, image resolution at 0.125–0.250 mm was used. The scans were analyzed using the inbuilt software (i-Dixel one volume viewer 1.7.0) in the coronal, sagittal, and transvers planes. The long axis of each tooth was determined and cross-sectional images from coronal to apical of roots were evaluated for root fusion and C-shaped canal configuration by rolling the tool bar from the pulp chamber to the apex. All scans were evaluated separately by two endodontists and any disagreement was discussed until a consensus was reached.

The criteria for defining C-shaped canal were adopted and modified from Jo HH et al. [9] and Martins et al. [16], when any arbitrary cross-section presents a C-shaped root canal configuration. Based on fused canal number and canal merging position, 2 major types and 5 subclasses of C-shaped canal system of maxillary molars were recorded. The major types were classified based on the number of fused root canals. The subclasses were classified by the sequence of fused canals. Abbreviation of capital letters were briefly used, such as, B, buccal canal; MB, mesiobuccal canal; DB, distobuccal canal; P, palatal canal. The ‘-’between capital letters means fusion of canals. (e.g., MB-P represents the C-shaped canal with fused mesiobuccal root canal and palatal root canal.

Age groups were divided as follows: ≤ 20, 21–30, 31–40, 41–50, ≥ 51 years. The incidence of bilateral symmetry was also recorded.

### Statistical analysis

Kappa test was used for intrarater reliability. The interobserver reliability was high for all evaluated teeth regarding C-shaped canals identification (Cohen’s Kappa > 0.91) and their classification (100% of agreement). Chi‑square analysis was performed to examine the relationships of C-shaped canal, according to age, sex, locations and bilateral symmetry. The Z‑test was used to compare proportions between independent groups. The differences were considered significant when *P* < 0.05. Statistical analysis was performed using SPSS (Version 22.0, SPSS Inc., Chicago, IL, USA) software.

## Results

### Prevalence of C-shaped canals in MFMs and MSMs

We first looked at the overall presence of C-shaped canals in MFMs and MSMs. As shown in Table 1, of the 1488 MFMs and 1547 MSMs examined, 89 teeth (2.93%) have C-shaped canal configuration. In MFMs, C-shaped configuration was noted in 8 teeth (0.54%), whereas in MSMs, 81 teeth (5.24%) present C-shaped configuration. The occurrence of C-shaped canals in MSMs was significantly higher than that seen in MFMs (*P* < 0.001). Root fusion may result in root canal merging. C-shaped canal is one type of the merging canal. We then looked at the presence of C-shaped canals in the fused roots. In MFMs, 61 fused roots (4.10%) were noted, and C-shaped canals were seen in 1.33% of these teeth. In MSMs, 539 teeth (34.84%) have fused roots and 13.50% of them present C-shaped canals. The prevalence of C-shaped canals in MFMs and MSMs with fused roots are significant higher (*P* < 0.05) than in those teeth without fused roots. These results indicate that in this Southwest Chinese population, C-shaped canals are more prevalence in MSMs than in MFMs, especially in MSMs with fused roots.

**Table 1:** Absolute counts and prevalence of C-shaped canal in MFMs and MSMs

	C-shaped canal (*n* %)		
	YES	NO	Total
Teeth			
MFMs			
Within first molar	8 (0.54%)***	1480 (99.46%)	*n* = 1488
With fused root	8 (1.33%)	53 (8.83%)	*n* = 61
% within all teeth	0.26%	48.76%	
MSMs			
Within second molar	81 (5.24%)***	1466 (94.76%)	*n* = 1547
With fused root	81 (13.50%)	458 (76.33%)	*n* = 539
% within all teeth	2.67%	48.30%	
Total			*n* = 3035
with fused root	89 (14.83%)	511 (85.17%)	
% within all teeth	2.93%	97.07%	

Fused root: *n* = 600

***P < 0.001

### Characteristics of C-shaped canals in MFMs and MSMs

**Fig. 1:**
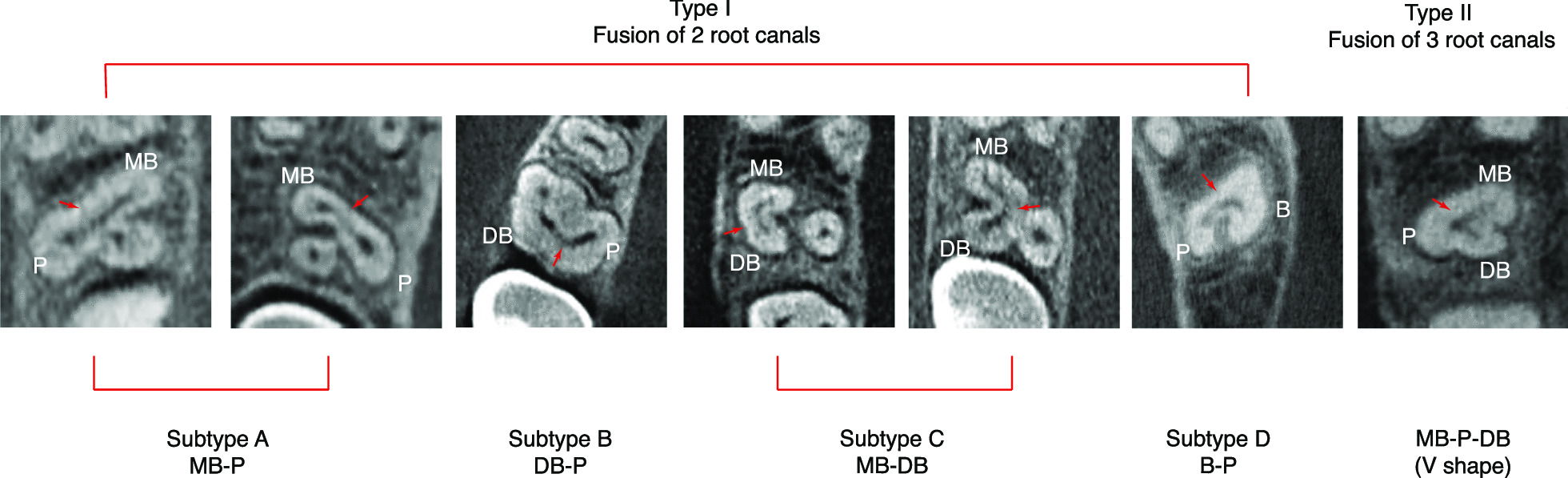
Represent CBCT images of different types of C-shaped root canal in MFMs and MSMs. Two major types and 5 subtypes of C-shaped root canals were noted in MFMS and MSMs. MB-P: fusion of mesiobuccal and palatal canals; DB-P: fusion of distobuccal and palatal canals; MB-DB: fusion of mesiobuccal and distobuccal canals; B-P: fusion of buccal and palatal canals; MB-P-DB: fusion of mesiobuccal, palatal and distobuccal canals

Maxillary C-shaped molars have low prevalence but high anatomic complexity [5]. To understand the characteristics of these C-shaped canals, CBCT images were further evaluated based on the classification previously described [9] [16] with minor modification (Fig. 1). Type I: C-shaped root canal was defined as fusion of 2 root canals (tooth with 2 roots or 3 roots) in C shape. It was further classified into 4 subtypes based on the canals involved. Type II: C-shaped root canal was defined as the fusion of 3 root canals (tooth with 3 roots) in C shape. As shown in Table 2, the most common type of C-shaped canals is type I subtype C (MB-DB). All the MFMs with C-shaped canals present as Type I subtype C (MB-DB). In addition, this subtype was observed in 69.14% MSMs with C-shaped canals. This difference was statistically significant (*P* < 0.001). Type II C-shaped canals are the second common in the examined MSMs (16.05%), followed by Type I subtype B (DB-P) (7.41%), Type I subtype A (MB-P) (4.94%), and Type I subtype D (B-P) (2.47%).

**Table 2:** Type and number of C-shaped root canal in maxillary first and second molars

Type of C-shaped root canal			
		MFMs(*n* = 1488)	MSMs(*n* = **1547**)
Type I Fusion of 2 root canals			
	Subtype A: MB-P	0 (0.00%)	4 (4.94%)
	Subtype B: DB-P	0 (0.00%)	6 (7.41%)
	Subtype C: MB-DB	8 (100%)***	56 (69.14%)***
	Subtype D: B-P	0 (0.00%)	2 (2.47%)
Type II Fusion of 3 root canals	MB-P-DB (V shape)	0 (0.00%)	13 (16.05%)

MB-P: fusion of mesiobuccal and palatal canals; DB-P: fusion of distobuccal and palatal canals; MB-DB: fusion of mesiobuccal and distobuccal canals; B-P: fusion of buccal and palatal canals; MB-P-DB: fusion of mesiobuccal, palatal and distobuccal canals

***P < 0.0001, compared to the other types

### Gender differences in the prevalence of C-shaped canals in MFMs and MSMs

Of the total 89 teeth with C-shaped canals, 31 were found in male (*n* = 1201) with a prevalence of 2.58%, and 58 were found in female (*n* = 1834) with a prevalence of 3.16% (Table 3). However, the differences in the prevalence between male and female patients was considered not significant (*P* > 0.05).

**Table 3:** Absolute counts and percentages of C-shaped canal between sexes and sides

C-Shaped canal	YES	NO
Sex		
Within male sex	31 (2.58%)	1170 (97.42%)
Within female sex	58 (3.16%)	1776 (96.84%)
Side		
Within right side	52 (3.37%)	1493 (96.63%)
Within left side	37 (2.48%)	1453 (97.52%)

### Presence of C-shaped canals in left or right MFMs and MSMs

Of the 89 teeth with C-shaped canals, 52 cases were from the right side (*n* = 1545) with a prevalence of 3.37%, and 37 were from the left side (*n* = 1490) with a prevalence of 2.48% (Table 3). No significant difference in the prevalence of C-shaped canals in left or right MFMs and MSMs were identified (*P* > 0 0.05).

### Prevalence of C-shaped canals in MFMs and MSMs in different age groups

As shown in Table 4, the 31–40 years age‑group showed the highest prevalence of C-shaped canals (4.09%), although no significant difference was noted among different age groups (*P* > 0.05).

**Table 4:** Absolute counts and types of C-shaped canal between age

Type of C-shaped root canal	Total(*n* = 3035) Age(years)				
	≤ 20 (*n* = 231)	21–30 (*n* = 808)	31–40 (*n* = 708)	41–50 (*n* = 659)	≥ 51 (*n* = 629)
Type I	0 (0.00%)	18 (2.23%)	25 (3.53%)	14 (2.12%)	19 (3.02%)
Type II	2 (0.87%)	2 (0.25%)	4 (0.56%)	4 (0.61%)	1 (0.16%)
Total	2 (0.87%)	20 (2.47%)	29 (4.09%)	18 (2.73%)	20 (3.18%)

### Incidence of bilateral presence of C-shaped canals in MFMs and MSMs

**Fig. 2:**
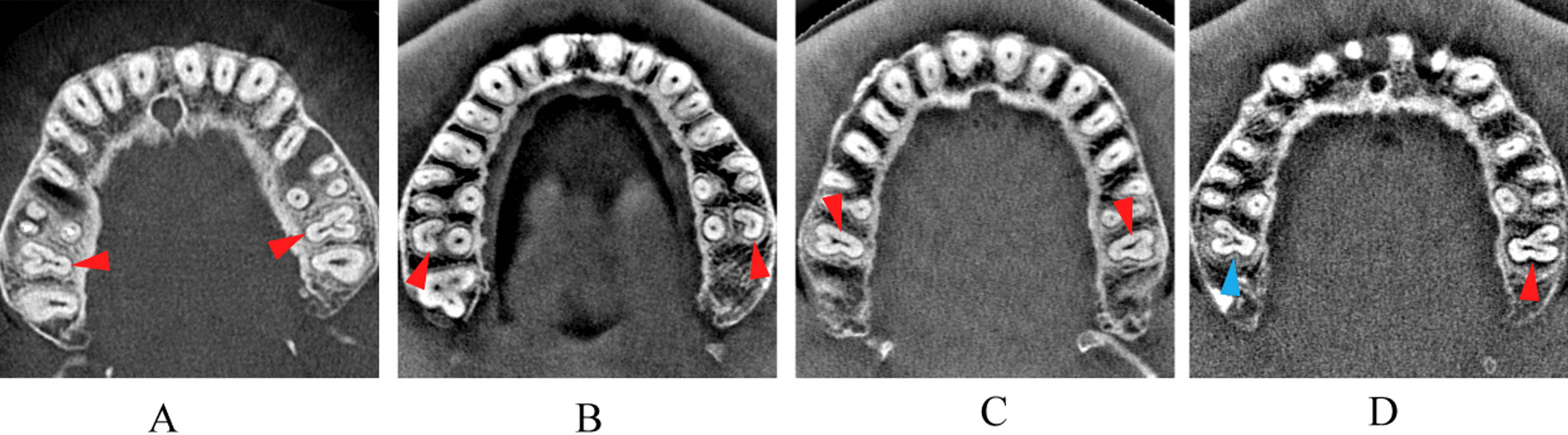
Represent CBCT images of bilateral presence of C-shaped canals in MSMs and MSMs. **A**, red arrow showed biliteral presence of Type II (MB-P-DB) C-shaped root canals. **B**, red arrow showed biliteral presence of type I subtype C (MB-DB) C-shaped root canals. **C**, red arrow showed biliteral presence of type I subtype A (MB-P) C-shaped root canals. **D**, red arrow showed the presence of Type I subtype B (DB-P) C-shaped root canals, and blue arrow showed the presence of Type II (MB-P-DB, V shape) C-shaped root canals

A total of 711 pairs of bilateral teeth (352 MFMs and 359 MSMs) were identified among the evaluated CBCT images from the 1508 patients (Fig. 2). Among them, 2 pairs of MFMs (0.57%) and 7 pairs MSMs (1.95%) present C-shaped canals, with a total incidence of 1.27% in maxillary molars. The same (Fig. 2A–C) or different (Fig. 2D) types of C-shapes could be seen in the bilateral symmetrical teeth. However, the main type of C-shaped canals among these bilateral symmetrical teeth was type I subtype C (MB-DB) (66.67%) (Fig. 2B).

## Discussion

Unusual root canal anatomy always poses a diagnostic and treatment challenge. In this study, we investigated the incidence and types of C-shaped root canals in the MFMs and MSMs in Southwestern Chinese subpopulation using CBCT. Different methods have been used to investigate the root canal morphology including canal staining and tooth clearing [17], periapical radiographs [18], and micro-computed tomography (micro-CT) [19, 20], all of which have some limitations. CBCT has been widely used in endodontic diagnose and treatment in recent years because of its higher accuracy, lower effective radiation doses compared with conventional CT and lack of specimen destruction [3]. In this study, we were able to obtain a large sample size from subjects who required radiographic examination by CBCT as part of their dental treatment.

In this study, the overall prevalence of C-shaped canal was 89 (2.93%) in MFMs and MSMs. Compared with previous studies performed in Korean (1.8%) [9], and Saudi Arabian (0.8%) [21] populations by using in vivo CBCT, our study identified a higher incidence of C-shaped canal. A Portuguese population study [16] that applied a methodology in line with the current research showed similar results (2.6%). Using an ex vivo evaluation method, a Brazilian study with 100 subjects [20] reported the prevalence of C-shaped canal in MFMs and MSMs to be 22%. The major drawback of this study is their limited sample size, which is doubtful if it adequately reflects the population. In our study, of the 600 fused roots, C-shaped canal was seen at 1.33% and 13.50% for MFMs and MSMs, respectively. However, C-shaped canals within fused roots were observed in 8.3% of MFMs and 5.1% of MSMs in a Saudi Arabian population [21]. The differences among these studies are possibly due to the differences in the ethnic background, sample size, study design (clinical or laboratory based), and the method of canal identification.

Some studies did not mention C-shaped canals but only reported merging canals within root fusion. By using micro-CT technology, Zhang et al. [19] identified a 35.4% prevalence of merging root canals within fused roots in a Chinese population. Another CBCT study of the native Chinese population revealed [22] that merging canals within fused roots were 4.5% in MFMs and 10.6% in MSMs. A Turkish population study [23] showed the presence of two‑merged canals in 31.52% and multiple merged canals in 8.48% of the MSMs. The root canal merging appears to be more common than C-shapes in fused roots. In our study, a large semilunar root canal shape that may represent a complete or partial root canal merging between 2 or 3 root canals was used to describe C-shaped canal in maxillary molars.

In the present study, we adopted and modified the criteria of Jo HH et al. [9] and Martins et al. [16] to define C-shaped canal in maxillary molars. Five different types of C-shaped molars were found by Martins et al. [16] and 3 different types and 6 subtypes of C-shaped molars was found by Jo HH et al. [9], which classified molars with fusion as fusion of entire or one-third of canals. In our study, we defined C-shaped canal when any arbitrary cross-section presents a C-shaped root canal configuration. C-shaped canals form an isthmus, which could become a clean-up blind spot and require the use of copious irrigation to clean. Therefore, identifying smaller C-shaped canals is clinically important. Based on fused canal number and canal merging position, 2 main categories and 5 subclasses of C-shaped canals were classified in our study. Several variations of C-shaped root canals in maxillary molars were reported earlier. C-shaped root canals of MB-DB was the most common type (32%) in a micro-CT study in maxillary molars in a Brazilian subpopulation [20], which was also a common type in our study. In addition, we showed that all C-shaped root canals in MFMs are Type I subtype C (MB-DB) (8 teeth), which was in agreement with the results reported in a Korean population [9]. However, another CBCT study in a Portuguese population showed that MB-P and DB-P were observed on MFMs [16]. In our study, merging of 3 canals on MB-P-DB (V shape) was only observed in MSMs. We did not see a DB-MB-P type as reported in a Korean population study [9], or a merging of two palatal canals into C-shape [24]. However, in a Turkish population study [23], no merging of multiple canals was observed. In our study, some subjects have four roots with 2 palatal roots, but the canals weren’t merged.

Regarding location, no significant difference in the prevalence of C-shaped canals was noted in right or left maxillary. This result are consistent with the findings of Mashyakhy M et al. in a Saudi Arabian Population [21]. In addition, C-shaped root canal configurations appear not to be related with gender, and same conclusion was reported by Tzeng LT et al. [25] regarding merging canals in a Chinese population. However, previous study [16] in a Portuguese population showed that C-shape is more common in maxillary molars of females. The differences in sample sizes or geographic regions might contribute to the variations in these results.

Regarding age, our study showed that the incidence of C-shaped canal has no relationship with age. Previous studies [9, 11] also revealed no relationship between age and the incidence of C-shaped canal. A Turkish population study [23] showed that the highest root fusion rate was at 41–50 age group. In our study, the 31–40 age group showed the highest C-shaped canal rate (*P* > 0.05). However, the types of C-shaped canal are different in different age groups (*P* < 0.05).

In the present study, we observed a low incidence of bilateral symmetry of C-shaped canals in maxillary molars. Among 352 pairs of MFMs and 359 pairs of MSMs, 0.57% first molars (2 teeth) and 1.95% second molars (7 teeth) were bilaterally symmetrical. Previous studies in Portuguese [16], Korean [9], Saudi Arabian [21] and Chinese populations [10, 19, 22] reported no bilateral symmetry of C-shaped canals in maxillary molars. A recent work in a Saudi Arabian Population showed only 1 patient appeared to have 2 MFMs (right and left) with C-shaped canals [21]. Therefore, the presence of C-shaped canal in one side of the maxillary molars may not imply its presence in the other side.

In cases of maxillary molars with C-shaped root canal system configurations, use of operating microscope, instrumentation with anti-curvature technique and irrigation with sodium hypochlorite is recommended due to the presence of isthmus and some dentinal thinness walls in C-shaped canals [26]. A study on the Self-Adjusting File system found that this instrument adapted itself to the root canal anatomy and was more effective in shaping the C-shaped canal compared with a conventional rotary system [27], which could enhance the success of root canal therapy.

In this study, some types of C-shaped canals reported in other studies were not found in the present study (e.g. MP-DP, tooth with 4 root canals) [9]. In addition, the number of recruited patients under age of 20 were much fewer than the other age groups and the incidence and type of C-shaped canals was greatly different from the other age groups, which may affect the final results. Future studies should include more subjects and similar numbers of subjects from different age groups.

## Conclusions

C-shaped root canals are more frequently present in MSMs than in MFMs in the Southwestern Chinese population, especially in MSMs with fused roots. Type I subtype C (MB-DB) C-shaped root canals are the most common subtype seen in this population.

## Data Availability

The datasets used or analyzed during the current study are available from the corresponding author on reasonable request.

## Abbreviations

MFMs: Maxillary first molars
MSMs: Maxillary second molars
MB: Mesial-buccal
MD: Mesial-distal
DB: Distal-buccal
P: Palatal
B: Buccal
CBCT: Cone-beam computed tomography scanning
micro-CT: Micro-computed tomography
